# Formation and In Vitro Simulated Digestion Study of Gelatinized Korean Pine Seed Oil Encapsulated with Calcified Wax

**DOI:** 10.3390/molecules28217334

**Published:** 2023-10-30

**Authors:** Peng Wang, Honglu Wang, Yanli Hou, Jingyi Wang, Yue Fan, Na Zhang, Qingqi Guo

**Affiliations:** 1 College of Life Science, Northeast Forestry University, Harbin 150040, China; wangpeng981101@163.com (P.W.); w172156786@126.com (H.W.); houyanli171@163.com (Y.H.); 18846000208@163.com (J.W.); fy1449945467@163.com (Y.F.); 2 College of Food Engineering, Harbin University of Commerce, Harbin 150028, China

**Keywords:** natural wax, calcified wax, Korean pine seed oil gel, microstructure, in vitro digestion, lipolysis rate

## Abstract

Natural waxes have demonstrated exceptional potential as oil gels for saturated and trans fatty acids, but their application has been limited by issues such as temperature sensitivity, lack of stability and durability, and compatibility. In this study, three types of wax (Beeswax (BW), Rice bran wax (RBW), and Carnauba wax (CW)) were combined with calcium hydroxide to produce calcified wax. The calcified Korean pine seed oil gel obtained by heating and stirring with Korean pine seed oil is responsive to temperature and has environmental adaptability. The effects of critical gel concentration, temperature regulation, texture properties, microstructure, oil-holding capacity, and FT-IR on the quality parameters of oil gel were investigated. Additionally, an in vitro digestion model was developed to comprehend the decomposition rate of fat during gel structure digestion and transportation. The results demonstrated a close correlation between the critical gelation concentration and calcium ion content. Furthermore, after calcification, the hardness followed the order BW > CW > RBW. Moreover, there was an approximate 10 °C increase in wax melting point. Conversely, BW:Ca exhibited the lowest oil leakage. The microstructures revealed that the oil gels formed post-wax calcification exhibited similar fractal dimension (Db) values (<7 μm), and the intermolecular forces were characterized by van der Waals forces, which were consistent with those observed in the non-calcified group. In conjunction with the vitro digestion simulation, our findings demonstrated that RBW and CW oil gels gradually released 20%, 35%, and 35% of free fatty acids (FFA) within the initial 30 min of intestinal digestion. Importantly, the FFA release rate was significantly attenuated, thereby providing a foundation for developing wax-based gel processed foods that facilitate gentle energy release benefits for healthy weight management.

## 1. Introduction

Natural wax is a material that comes from plants or insects and is a renewable resource [1,2,3]. The main constituents of the substance predominantly include wax esters, fatty alcohols, fatty acids, and n-alkanes, as indicated by previous studies [4,5,6,7]. Due to its cost-effectiveness and exceptional gelling properties [8,9,10], Beeswax (BW), Rice bran wax (RBW), and Carnauba wax (CW) have found extensive applications in the cosmetic, pharmaceutical, and food industries [11,12,13]. In the realm of food science, the utilization of BW, RBW, and CW as fat substitutes for oil gel preparation has emerged as a novel and viable approach. Numerous published studies have investigated their mechanisms and practical applications [6,14,15,16]. For instance, wax-based oil gels are employed in confectionery fillings and chocolate formulations to reduce saturated fatty acid intake [9,17]. The incorporation of waxes not only facilitates the formation of a well-structured gel network with oils but also retards oxidation processes [11,18], thereby extending the shelf life of oils. Furthermore, the texture and sensory attributes of oil gels can be modulated by waxes to impart a plumpness and silky touch to products while enhancing user satisfaction [19,20,21]. Additionally, the porous structure endowed by these waxes enables control over the release rate and permeability of active ingredients [22,23], reduces volatilization or migration issues associated with active compounds, and prolongs their efficacy period [12,13,20].

Korean pine seed oil (KPSO) is a vegetable oil that is typically obtained through cold pressing or solvent extraction of the oil found in the seeds [24,25]. KPSO is abundant in unsaturated fatty acids, vitamin E, minerals [25], and various other beneficial nutrients that contribute to reducing blood pressure, enhancing blood circulation, safeguarding liver health, and facilitating cellular repair [26]. It is feasible to formulate Korean pine seed oil gel using natural wax [9,18], thereby eliminating trans fats and enabling the preparation of edible grease gels that align better with the health-consciousness of modern consumers. However, the existing literature suggests that these natural waxes exhibit temperature sensitivity [23], resulting in hardening and embrittlement at low temperatures [9] and softening and melting at high temperatures. These characteristics may result in easy decomposition or a reaction with other compounds under specific conditions like elevated temperature or humidity [27], thereby adversely affecting their original properties. Solubility and compatibility rely on their chemical structure and molecular size [28,29,30]. Certain natural waxes may not be compatible with specific solvents or other substances; moreover, different types of natural waxes may exhibit variations in composition and properties, which pose challenges for maintaining consistency and repeatability during application [7]. As most wax-based gel droplets tend to be large and unstable, they are susceptible to mechanical forces, which present difficulties for subsequent processing steps as well as application. Further exploration is required for modifying and preparing stable wax-based gels.

The digestion of oil gel is an interfacial process, which is facilitated by the absorption of lipase onto the interface of emulsified fat droplets [31,32]. Given that enzymes are typically present in excess relative to their substrates [33], the primary mechanism regulating fat digestion involves controlling the ability of lipase to bind to the interface of emulsified fat droplets through inhibition of enzyme activity [34,35]. This can be through interface composition or area [36] or through packaging techniques [37]. The digestion of wax-based oil gels relies on factors such as fatty acid diversity, structure and location, physical state of lipids, and properties of the surrounding food matrix or interfacial film [38]. Both in vitro and in vivo studies have consistently demonstrated slower digestion rates for solid fats compared to higher rates observed for liquid fats [32,39]. By simulating physiological conditions within the human gastrointestinal tract, including different pH environments, enzyme activities, and digestion rates [40,41], an in vitro simulated digestion research model aids in comprehending properties, stabilities, and behaviors specific to KPSO gel [42]. It also helps elucidate its performance within the oral cavity, stomach, and small intestine regions, encompassing disintegration rate, release rate, and stability aspects [24,43]. Furthermore, evaluating taste perception along with stability characteristics is for the purpose of developing healthier and more efficient products. Detailed understanding regarding structural changes (microstructure, molecular interactions) during digestion provides profound insights into the digestive behavior exhibited by oil gels [44,45,46]. Therefore, this study primarily aims to explore and discuss KPSO gel constructed from calcified wax concerning its physical state as well as digestive functionality.

**Figure 1:**
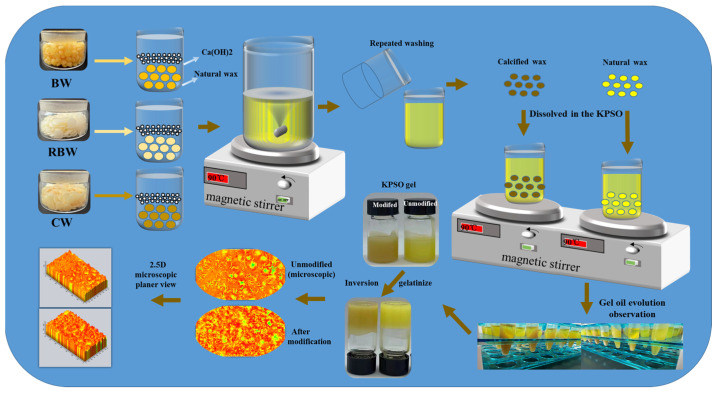
Schematic diagram of preparing KPSO gel by calcified wax.

In this study, three types of calcified waxes were prepared (Figure 1). Calcified Beeswax, Calcified Rice bran wax, and Calcified Carnauba wax, along with wax bases (BW, RBW, CW), were utilized for the fabrication of KPSO gel to assess in vitro simulated fatty acid digestibility. Various analytical techniques, including polarized light microscopy, Fourier transform infrared spectroscopy, particle size analysis, and TPA measurements, were employed to investigate the microstructure of different calcified wax-based oil gels as well as the impact of temperature intervention on their macro properties. Furthermore, in vitro digestion models (oral, gastric, intestinal) were used to assess bioaccessibility and kinetics. These insights into the application of KPSO gel offer guidance for optimizing and enhancing edible wax bases.

## 2. Results

### 2.1. Physical Characterization of KPSO Gel

**Figure 2:**
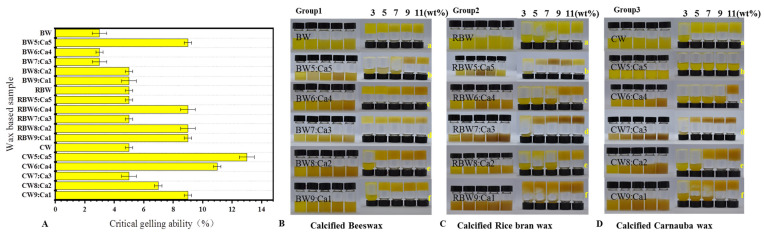
KPSO gel Critical gelling concentration (C*) (**A**); calcified wax KPSO gel BW (**B**); RBW (**C**); CW (**D**). ((wt%) indicates the weight percentage).

The efficiency of the three calcified wax combinations in forming gels with KPSO was evaluated by referencing the Winkeler-Moser method [11] to determine the minimum gel requirement. The critical gel concentration (C*) is the point where no visible flow occurs in the bottle, according to previous studies [11,12,13]. As depicted in Figure 2A, BW, RBW, and CW exhibited critical gel formation concentrations of 3%, 5%, and 5 wt%, respectively. It was observed that a gel could be formed at a low melting point concentration of 3 wt% for BW; however, RBW and CW failed to form gels at this concentration. Interestingly, an increase in melting point did not result in a gradual decrease in C*, suggesting that C* is influenced by wax composition [28,47,48].

The Critical gelling concentration (C*) of oil gels with varying proportions of calcium wax exhibits significant variations. In Figure 2A, a gel can be formed with 3% BW:Ca (9:1), while RBW:Ca and CW:Ca (9:1) require 9 wt% C* for gel formation. The addition of calcium ions leads to an increase in C*. Previous studies have indicated that the choice of solvent also influences C* [3,18,19]. Solvents capable of forming well-structured crystal networks with wax tend to exhibit lower C*, indicating higher oil-binding capacity [9]. To ensure experimental consistency, this study conducts experiments based on KPSO. According to the findings of Figure 2B–D, BW:Ca demonstrates a robust gelation ability and effective collaboration with BW and Ca^2+^, followed by RBW:Ca. However, CW:Ca exhibits inferior performance in this regard. As the concentration increases, the stability of oil gels formed by these three types of calcified waxes gradually improves. Based on these results, it is observed that at 3 wt% concentration, except for BW:Ca ratios of 6:4 and 7:3, none of the calcified wax groups can independently form a stable oil gel network; instead, they remain in a liquid state. At an 11 wt% concentration level, stable gels can be produced except for CW:Ca ratio of 5:5. Considering different waxes have distinct C* concentrations, we selected an 11 wt% concentration to further evaluate the gel behavior of various calcified waxes to ensure consistency among variables [12,18,21].

The mechanical property analysis presented in Table 1 is consistent with the findings depicted in Figure 2B–D. However, it should be noted that while calcified wax with varying proportions can form oil gel, statistical analysis reveals a significant disparity in hardness between natural wax and calcified wax due to the complexation involving calcium wax [27]. The BW:Ca ratios of 6:4 and 7:3 oil gels exhibit significantly higher hardness compared to the BW:Ca ratios of 5:5, 8:2, and 9:1 oil gels, with notable variations observed among different ratios. Notably, the oil gels containing higher RBW:Ca ratios of 7:3, 8:2, and 9:1 demonstrate enhanced hardness in comparison to the oil gels with lower ratios (6:4), while calcification leads to a significant reduction in CW gel hardness. Specifically, BW6:Ca4 exhibits the highest gel hardness (>530 g), followed by RBW:Ca with a ratio of (9:1) at a maximum gel hardness of 365 g and CW:Ca with a ratio of (7:3) at approximately 126 g. Additionally, this study demonstrates an inverse correlation between the Ca^2+^ ratio and the minimum concentration required for gelation. Only combinations exceeding the critical gel concentration can achieve a stable gel form [12,13,48]. Differences were observed in the textural properties of calcified wax-based oil gels at various concentrations (*p* < 0.05), with hardness, mastication, and adhesivity decreasing as Ca^2+^ levels increased. Moreover, notable variations in certain textural properties were observed among different types of calcified wax-based oil gels. Table 1 reveals that beyond a certain threshold of wax base concentration, there are no significant differences in partial viscosity, cohesion, and elasticity. Further comparison of texture results indicates gel exhibits the highest comprehensive hardness, adhesive force, and adhesive properties, followed by RBW:Ca and CW:Ca. CW displays superior cohesiveness and chewability, while RBW demonstrates greater elasticity. The texture properties of the BW suggest its ability to rapidly form a gel state and effectively cure KPSO.

### 2.2. Influence of Temperature on Gel Strength of KPSO Gel

**Figure 3:**
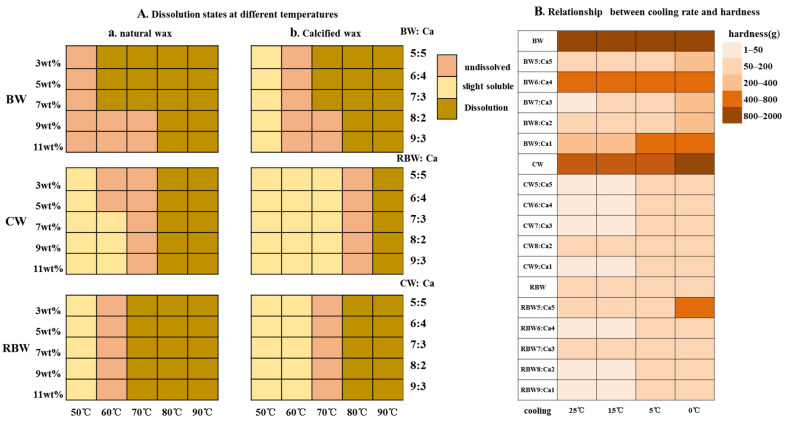
Natural wax (**a**) and calcified wax (**b**) exhibited distinct dissolution behaviors at varying temperatures in KPSO solution (**A**). The relationship between different cooling rates and hardness was investigated (**B**).

Prior to conducting the gel strength test, oil samples and wax were heated on a magnetic stirrer at temperatures of 50, 60, 70, 80, and 90 °C for 30 min to ensure complete dissolution of all wax components in the oil. The results depicted in Figure 3(Aa) demonstrate that BW exhibits slight solubility at 50 °C, with complete dissolution of the wax base as the temperature increases. However, both RBW and CW did not dissolve at 50 °C. As the temperature gradually increased, it dissolved at 70 °C while RBW dissolved at 60 °C. After calcification, as depicted in Figure 3(Ab), the melting points of BW:Ca, RBW:Ca, and CW:Ca increased to 60 °C, 70 °C, and 80 °C, respectively, exhibiting an approximate elevation of 10 °C for all samples. The observed increase in dissolution temperature signifies enhanced wax stability, which aligns with previous findings reported by Huang et al. [27]. Subsequently, the specimens were static cooling at temperatures of 0, 5, 15, and 25 °C for a duration of 1 h before being retrieved and left temperature for a period of 48 h. These changes in hardness were measured [3].

The relationship between cooling temperature and hardness was evaluated using KPSO gelation, depicted in Figure 3B. To maintain a consistent solid fraction and wax base concentration of 9%, the oil phase was cooled at temperatures of 0, 5, 15, and 25 °C for a duration of 1 h. It was observed that an increase in the Ca^2+^ ratio led to higher nucleation rates, resulting in the formation of numerous smaller crystals during the cooling process. Consequently, this led to varying degrees of reduction in hardness [3]. Notably, the highest and lowest levels were observed under different cooling conditions: at 0 °C (BW: 1686 g; CW5:Ca5: 34 g), at 5 °C (BW: 1652 g; CW5:Ca5: 24 g), at 15 °C (BW: 1531 g; CW6:Ca4: 18 g), and at 25 °C (BW: 1479 g; CW6:Ca4: 14 g). Furthermore, it can be inferred that CW:Ca exhibited lower hardness due to disruption caused by Ca^2+^ on its crystal structure, leading to inconsistent crystallization. Additionally, since Ca^2+^ possesses surface-active properties that reduce the interface between the oil phase and wax phase [9], it aids in solidifying the mixture after homogenization. 

### 2.3. KPSO Gel Oil-Binding Capacity and FT-IR Scanning

**Figure 4:**
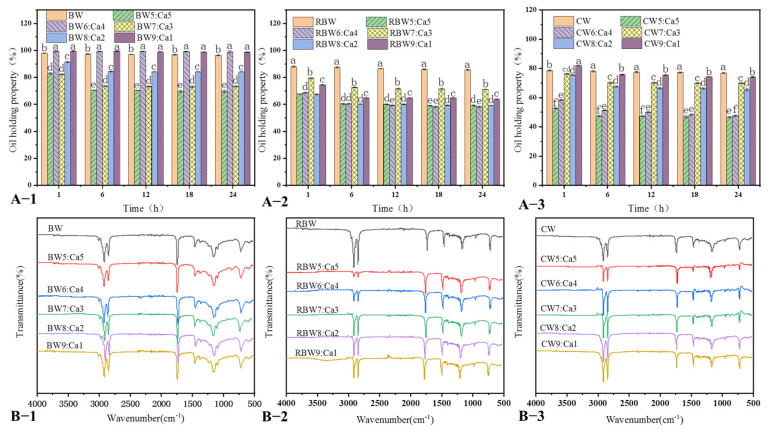
Oil-binding capacity of KPSO gel (**A-1**–**A-3**) (Note: different letters indicate significant differences in data); FT-IR (**B-1**–**B-3**).

**Figure 5:**
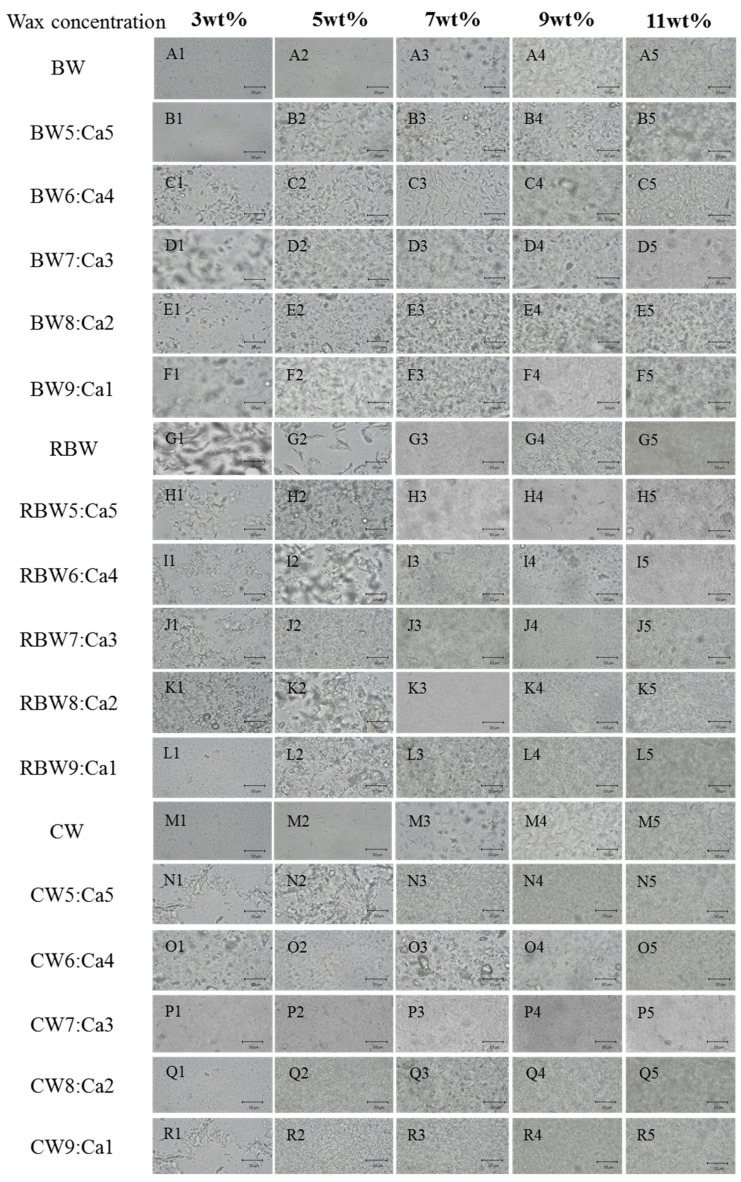
Optical microscope images of various calcified wax KPSO gels (3, 5, 7, 9, and 11 wt% is the mass ratio of added wax concentration) with a scale of 10 μm. (Note: all gel observations were conducted at a magnification of 400×).

Figure 4A shows the oil-binding capacity (OBC), measured as the mass of the initial oil gel minus the mass percentage of the spilled oil over time [21]. Both untreated wax-based and calcified wax-based mixtures exhibited moderate oil leakage, reaching a steady state after 12–18 h. Figure 4(A-1) illustrates BW:Ca ratios of 5:5 (70%), 6:4 (99%), 7:3 (73%), 8:2 (84%), and 9:1 (98%) with corresponding oil leakage of 0.2% to 35%. In contrast, Figure 4(A-2) depicts RBW:Ca ratios of 5:5 (60%), 6:4 (3(71%), (59%), and 9:1 (64%). The oil gels based on Figure 4(A-3) CW:Ca 5:5 (46%), 6:4 (47%), 7:3 (70%), 8:2 (66%), 9:1 (74%) exhibited a higher degree of oil loss, with approximately 30–50% reduction in oil content compared to the untreated wax base exception of BW:Ca (6:4, 9:1), the oil retention rate of the calcified wax-based mixture decreased by 1–50%, which may be attributed to a reduction in gel oil absorption rate after the calcification of different wax groups. BW:Ca demonstrated strong gel ability, indicating that high levels of wax esters and hydrocarbons can form a robust network structure [18]. Conversely, RBW:Ca and CW:Ca exhibited a weak network structure due to the absence of long-chain components, resulting in low oil-binding capacity [12,13]. Interestingly, despite CW having the highest melting point and higher wax ester content, the presence of higher fatty alcohol content might hinder crystallization and lead to reduced oil-binding capacity [13]. The oil-holding capacity of all calcified wax oil gels remained stable after 24 h; compared to BW and RBW cases after calcification, the CW-dominated case demonstrated significantly higher rates of oil loss. Figure 5 illustrates that BW forms randomly arranged, elongated, needle-like crystals, which increase the surface area (particularly in three dimensions), thereby enhancing its ability to bind with a network structure, and oil-holding capacity has been established [11].

The intermolecular forces of three Wax:Ca (5:5, 6:4, 7:3, 8:2, 9:1) oil gels were determined using *FT-IR* (Fourier transform infrared spectrometer) spectroscopy, and the results are presented in Figure 4B. Three distinct bands were observed corresponding to the stretching vibrations of OH groups, carboxyl groups, and CH_3_ and CH_2_ groups, respectively. It is evident that the peak in the range of 1000–1200 (cm^−1^) can be attributed to C-O stretching vibration, while the characteristic peak related to ester bond formation appears near 1743 (cm^−1^) as a result of C=O stretching vibration [39]. Additionally, a weak absorption peak around 3011 (cm^−1^) was observed in KPSO gels, which corresponds to an overtone region of the C=O stretch; however, no characteristic OH peak was detected within the range of 3300–3400 (cm^−1^). Winkler-Moser et al. proposed that the solve-gel factor binding of long-chain fatty alcohols in CW disrupted bonding, resulting in diminished gel capacity [11]. Furthermore, the presence of C-H at 1743 and 1462 (cm^−1^) is associated with the bending vibration of the CH_3_ and CH_2_ groups. The absorption peaks at 1095–1165 (cm^−1^) correspond to the stretching vibrations of C-O-C and C-O-H, respectively [20]. The peaks observed near 2852 and 2916 (cm^−1^) are attributed to the stretching vibrations of CH_3_ and CH_2_, indicating the presence of unsaturated fatty acid chains within the gel structure. It has been previously reported that a shift in absorption peak positions for symmetric and antisymmetric CH_2_ stretching vibrations can be attributed to van der Waals interactions [6]. Untreated BW and CW samples exhibited significantly higher values compared to BW:Ca and RBW:Ca after calcification at 2956–2982 (cm^−1^); however, CW values were lower, possibly due to enhanced binding forces between CW and KPSO following calcification. These results demonstrate that wax-based oil gels with a higher content of unsaturated fatty acids possess more methyl groups exhibiting asymmetric stretching vibrations, which may arise [22,23]. Another explanation could be that the distorted spatial structure also affects electron absorption capacity in CO double bonds, leading to variations in methyl symmetric stretching vibrations [29].

### 2.4. Microstructure Observation of KPSO Gel

Polarized micrographs (Figure 5) were employed to compare and observe the microstructure of calcified wax-based oil gels. The results demonstrated that oil gels with varying concentrations of calcified waxplets, thereby maintaining stability. By promoting interfacial interactions and crystal network formation [18], these exhibited improved collision resistance, coalescence stability, and overall performance enhancement. The acicular BW crystal network structure exhibited a high density with well-ordered crystals, while RBW crystals formed clusters with larger particle sizes. Conversely, the dendritic CW structure resulted in gap crystals [48], which impeded lateral growth and compromised structural integrity.

Wax ester is the basis of oil gel formation, and the difference in wax base concentration directly leads to the difference in microstructure. Figure 5 (A1–A5, G1–G5, M1–M5) shows that the larger wax esters tend to crystallize at the interface, while the smaller wax esters tend to crystallize in the bulk phase. To achieve effective coating, it is crucial for adsorbed particles at the interface to have a much smaller diameter than that of droplets [11,23,27,49,50]. Generally speaking, smaller crystals are more easily adsorbed at the interface [9]. Furthermore, crystal shape also influences their presence at the interface; spherical crystals are more likely to be found due to their superior wettability compared to other shapes [23]. Figure 5 (B–F: BW:Ca; H–L: RBW:Ca; N–R: CW:Ca) demonstrates that calcium interface wax-based crystallization stabilizes calcified wax gel formation. The prepared calcified wax oil gel exhibits small-sized wax crystals because Ca^2+^ significantly inhibits crystal growth. The dispersion of the oil phase also influences the crystallization behavior of wax [1,11]. The presence of Ca^2+^ in calcified wax increases the average distance between wax molecules and crystals, leading to an enhanced diffusion rate and increased crystallization rate. Moreover, compared to non-calcified oil gels, the crystal density is reduced in calcified wax-based oil gels. This indicates that crystals are evenly distributed in calcified wax-based oil gels than in non-calcified acicular crystal structures; however, when wax crystals aggregate into large flocs, it becomes challenging to construct a network independently [18]. Therefore, they can enhance their structure by adsorbing oil droplets [21]. In other waxes that easily form crystal networks, CW flocculent is more prone to adsorption at interfaces due to its deposition on droplet surfaces [21,49], resulting in relatively dense interfacial terms of macroscopic properties. Oil gels prepared with decreasing calcified ratios (7:3, 8:2, 9:1) exhibit higher hardness and recovery rates among all tested samples. This confirms their superior crystal network strength and stability against mechanical forces compared to other oil gels, as supported by previous data [23]. In summary, Ca^2+^ plays a significant role in determining the structure and mechanical properties of oil gels.

The fractal dimension (Db) characterizes the spatial distribution of fat crystal particles, and the size of oil droplets is a crucial indicator for evaluating the oil gel [11]. Generally, a higher Db corresponds to a more ordered crystal structure [22]. As depicted in Figure 5, distinct waxes exhibit significantly diverse microstructures. Table 2 demonstrates that the KPSO gels employing calcified wax are BW:Ca > RBW:Ca > CW:Ca, respectively. Consistent with the findings in Section 2.3, oil-binding capacity, BW displays relatively high oil retention and a larger Db value, followed by RBW and CW. The Db values of all three wax bases decrease after calcification due to Ca^2+^ promoting interface crystallization through enhanced wetting behavior or heterogeneous nucleation at the interface. Ca^2+^ loading maintains an average diameter of 4 and 7 μm as it homogenizes with added Ca^2+^, resulting in better adsorption at the interface without interference from wax molecules, thereby forming smaller droplets.

### 2.5. Analysis of KPSO Gel by Digestive Model

**Figure 6:**
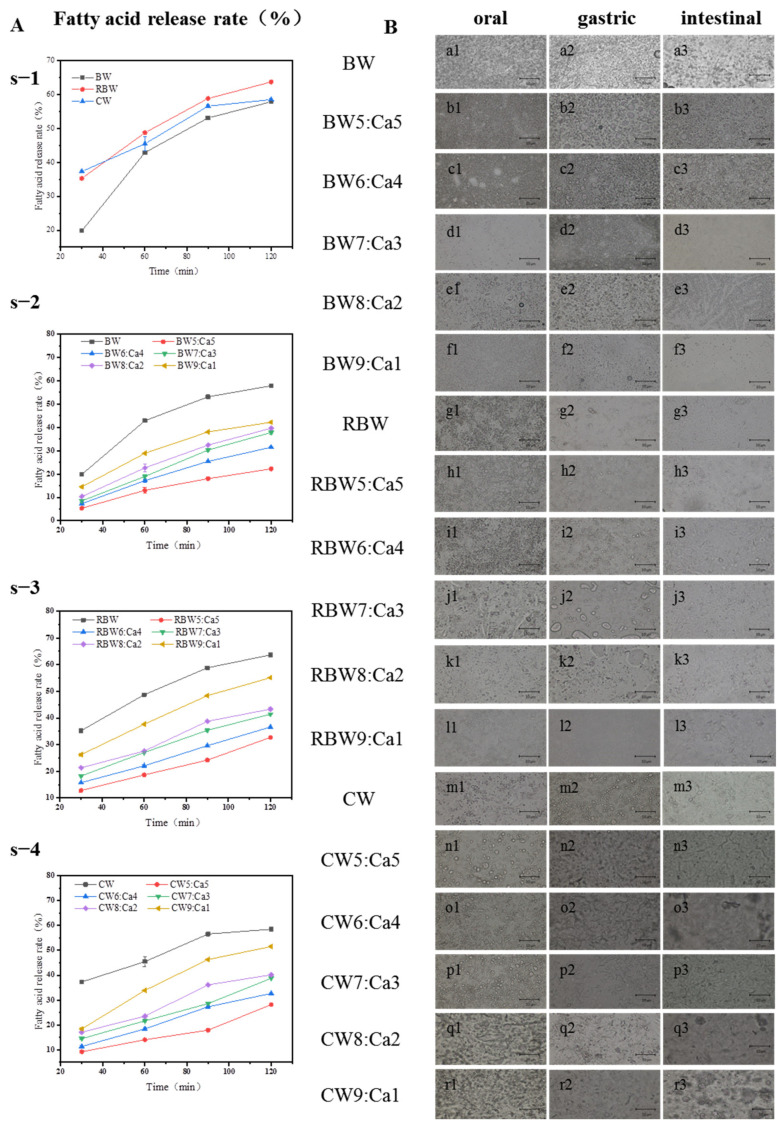
The fatty acid release rate of KPSO gel (**As-1**–**As-4**); oral digestion (1), gastric digestion (2), intestinal digestion (3). Light microscope image (**Ba1**–**Br3**) (Note: microscope magnification 400×, scale = 10 μm).

The fatty acid release rate values of the prepared oil gels were all <1, indicating a double digestion process: an initial rapid stage followed by a subsequent slow stage [36,41,42,43]. The high ratio of pancreatic lipase to available substrate leads to swift lipid digestion within the first few minutes of the wax-based fat content [37,40]. Figure 6A illustrates the deceleration of lipid digestion over time [31]. Importantly, although the oil gel partially limited lipid digestion, it did not modify the pattern of lipid digestion in vitro [44,45,46]. Overall, solid fats undergo slower digestion compared to liquid oils during intestinal processing, suggesting that calcified wax groups significantly influence the rate of lipid hydrolysis [38,39]. Through the protection provided by the fat crystals, the digestion of liquid oil entrapped within the network of fat crystals is delayed. The hydrolysis of oil gel lipid compounds exhibits a significant compositional dependence (Figure 6A). The release profiles of fatty acids from BW, RBW, and CW demonstrate that FFA content is gradually released during the initial minutes of intestinal digestion, with respective contents of 20, 35, and 35%. Subsequently, after 60 min, there is a decrease in lipid digestion rate for all three oil gels, resulting in approximately 15, 15, and 8% FFA release. These indicate that the presence of wax-based oil gels exerts an inhibitory effect on both lipid digestion rate and extent.

In the BW:Ca combination with a 9:1 mixture (Figure 6(As-1)), there was an initial sharp increase in content (reaching approximately 15% within 30 min), followed by a significant reduction in the release rate, resulting in about 40% of available fatty acids being released for hydrolysis after 30 min. In BW:Ca ratios of 5:5, 6:4, 7:3, and 8:2, the release rate of fatty acids was relatively slow, and about 20, 25, 30, 30% FFA was released within 2 h. This finding could be attributed to accessing ester bonds embedded within larger crystals compared to gaps present in fat crystal subcells and lamellae [37]. In contrast, the high mobility of BW molecules facilitates enzyme access to ester bonds [38]. During the initial stages of intestinal digestion, the rapid release of free fatty acids (FFA) is primarily attributed to liquid oil digestion [34]. With an increasing proportion of calcium wax, the rate of RBW:Ca digestion slows down more significantly in Figure 6(As-2). Initially, RBW5:Ca5 rapidly released 10% FFA within 30 min and then linearly increased to a content of 25% FFA after 2 h. For RBW:Ca ratios of 6:4, 7:3, and 8:2, approximately 30%, 35%, and 35% FFA were released within two hours, respectively. By increasing the calcium/wax ratio to 9:1, nearly one-fourth of available Lipid acidified within just half an hour, followed by another one-fourth being hydrolyzed within 90 min. While CW9:Ca1 released approximately 55% of FFA within 2 h, CW:Ca ratios of 5:5, 6:4, 7:3, and 8:2 exhibited release percentages of 20%, 25%, 30%, and 30% for FFA, respectively. This phenomenon can be attributed to the elevated melting point temperature and stable crystal structure of calcified wax, which effectively impedes KPSO diffusion and consequently retards lipid digestion [35].

Oral processing had minimal impact on droplet size in all three wax-based oil gels compared to the oil gel group (Figure 6(Ba1,Bg1,Bm1)), while the calcified gel group exhibited significant changes in droplet size (Figure 6(Bb1–Br1)). Gastric digestion led to a substantial increase in particle size of calcified wax-based gels (Figure 6(Bb2–Br2)). These enlarged oil droplets adsorbed with Ca^2+^ were formed due to pH variations, mechanical shear forces, and coalescence facilitated by pepsin digestion, confirming the effective protection of oil droplets from digestion-related decomposition by Ca^2+^. After nearly 120 min of intestinal digestion, the digested oil gel mainly consisted of coalesced oil droplets with an average diameter of approximately 10 μm and some dispersed flocs of oil drops. In the case of the calcified wax–oil gel mixture, gastric digestion for 60 min resulted in the appearance of large oil droplets and aggregates (Figure 6(Ba2–Bk2)), the precipitation of oil droplets, and partial mixing with dispersed wax base [41,42]. After intestinal digestion, spherical oil droplets are transformed into large, irregularly shaped fat aggregates with a diameter of approximately 10 μm due to the blocking effect of Ca^2+^, indicating partial coalescence of the oil gel facilitated by calcified wax. This phenomenon is attributed to the thesis of the interface layer surrounding the oil and subsequent exposure of Ca^2+^ on their surface, promoting this partial coalescence. Following 120 min of intestinal digestion, these large aggregates may undergo decomposition as a result of wax group hydrolysis. In oil gel containing a high proportion of Ca^2+^, gastric digestion for 60 min leads to the appearance of large oil droplets measuring tens of microns in diameter, which can be attributed to partial aggregation and flocculation/aggregation. However, in Figure 6B, where the proportion of Ca^2+^ gradually decreases over time during the gastric digestion process, aggregation also occurs due to exposure and subsequent partial coalescence or flocculation caused by CW hydrolysis at the oil–water interface. This indicates severe instability [24]. The formation of both large irregularly shaped oil droplets and small ones is observed consistently with our findings on oil retention.

Finally, in conjunction with the particle size data presented in Table 3, the digestion behavior of oil gels containing varying proportions of calcified wax was further investigated and discussed. It was observed that as the amount of calcified wax decreased, the gel combinations exhibited smaller and more uniform particles after oral digestion. Conversely, an increase in calcified proportion resulted in greater difficulty in mouth digestion. Hydrolysis of dispersed was found to be strongly influenced by the Ca^2+^ wax base, as evidenced by a decrease in the average lipid digestion rate with increasing Ca^2+^. Notably, the slow digestion of calcified wax can be attributed to limited lipase activity toward KPSO embedded within fat crystals. Furthermore, during gastrointestinal digestion, larger particles or flocculent aggregates gradually emerged [35], reaffirming that even at a 5:5 ratio of calcified wax composition in oil gels, dispersed wax exhibits a significantly faster digestion rate compared to dispersed Ca^2+^. At advanced stages of digestion, higher solid fat (calcified wax) appears to provide better protection for the present in KPSO [31,36,37]. This study enhances our understanding of the digestive behavior of both wax-based and calcified-wax-based oil gels while contributing valuable insights toward developing novel approaches for controlled and localized nutrient release applications.

## 3. Discussion

The oil-binding capacity of the calcified wax prepared in this study was significantly higher than that of the uncalcified wax [6,7]. This is due to the gradual increase in the injection degree with the addition of calcified wax. Consistent with Huang et al., calcium hydroxide reacts with fatty acid calcium, which greatly improves the hardness of calcified wax and further enhances gel crystal network degree of density and solid fat content [9,27]. However, the excessive addition of gels can lead to a high concentration of gels and an increased C* value for oil gels. When too much calcium hydroxide is added during calcification, intermolecular density decreases and becomes relatively loose, resulting in decreased hardness, opposite to what occurs when other types of gels are excessively added [26,34]. This may be because the natural wax base is reduced after calcification, which destroys its original structure, leading to microstructure recombination. The solubility and compatibility of gels depend on the miscibility and compatibility of the two substances, involving both physical and chemical forces [49,50]. Compared to uncalcified wax, oil gels constructed with calcified wax exhibit enhanced stability due to compatible modification between the wax base and Ca^2+^ [27]. In some studies, maintaining consistency during gel application is challenging due to variations in gel compositions [23], which significantly impacts the quality of gels. By adjusting the proportion of calcium wax, oil gels constructed with calcified wax successfully increase the availability of crystal materials. Needle or fibrous structures with crystal lengths ranging from 10 to 20 μm exhibit higher strength and hardness [28]. The volume and size of crystals in calcified wax can increase as the wax content increases [29]. These findings are consistent with previous studies by Winkler-Moser and Patel et al. [11,49]. Based on earlier research [10,30], fibrous structures have been identified as ideal for gelation owing to their high aspect ratio shapes that provide a larger surface area compared to low aspect ratio shapes such as spheres or platelets. The presence of a high surface area has been observed to promote the formation of microscopic elements, which are responsible for the elastic and solid states. Thereby facilitating enhanced interactions between organized molecules and the solvent [22]. Conversely, calcified wax diminishes crystal size due to a uniform distribution of crystal mass and small holes within the network [47]. Consequently, this necessitates highly contracted and zigzag pathways for volume migration in gels, resulting in reduced oil loss and slowed KPSO movement. These optimizations enhance the mechanics of gels, thereby holding significant implications for numerous applications.

Digestion of the oil gel made from calcified wax occurs in two stages: initially, a rapid stage, followed by a slow stage, which is consistent with the literature [31,35,38]. During the oral phase, the duration is brief and consequently limits the extent of digestive reactions. However, upon entering the gastric stage, the pH value of gastric juice does not decrease initially due to the buffering effect exerting fat [41]. Interestingly, as digestion progresses, there is an increase observed in the concentration of all free fatty acids. While gastric digestion causes a small amount of FFA, intestinal digestion results in a substantial release of FFAs. The FFA release curve of calcified wax gel showed a gradual increase trend during gastric fat dissolution, consistent with previous studies [38,39,41]. This similarity can be attributed to the protective effect of fat crystals on delaying the digestion of liquid oil trapped within. Furthermore, dilution with calcified wax raises the crystallization temperature and hinders KPSO diffusion, potentially slowing down lipid digestion [44,45]. These findings suggest that hydrolysis of calcified wax itself plays a significant role in the observed difference in acid release. The formation of large Ca^2+^-absorbing oil droplets within calcified wax-based gels is facilitated by pH changes, mechanical shear forces, and pepsin digestion [46]. These spherical oil droplets are large, irregular-shaped fat aggregates due to Ca^2+^ binding during intestinal digestion. It has been demonstrated that Ca^2+^ effectively protects oil droplets from breakdowns associated with digestion.

## 4. Materials and Methods

### 4.1. Materials

The Korean pine seed oil (KPSO) was obtained from the Key Laboratory of Forest Food Resources Utilization of Heilongjiang Province [24]. The Beeswax (edible grade) was purchased from Changge Yafei Bee Keeping Professional Cooperative, Rice bran wax (97% edible grade) was purchased from Huzhou Shengdao Ltd. (Changge, Huzhou, China), and carnitas wax (edible grade) was purchased from Brazil Foncepi Company (Piripiri, Brazil).

### 4.2. Chemicals and Reagents

α-amylase (S31302, ≥5 μ/mg solid), protease (S10028, 1:15,000), bile extract (S26985, 97%), and pancreatic enzyme (S10031, BR, 1:4000) were purchased from Shanghai Yuanye Biotechnology Co., Ltd. (Shanghai, China) All other chemicals used in this study were of analytical grade.

### 4.3. Preparation of Calcified Wax

Principle: The addition of calcium hydroxide initiates a reaction with the organic fatty acids present in the wax, resulting in a reduction of their content and subsequently lowering the acid value of the calcified wax. Additionally, the formation of fatty acid calcium salt contributes to an increase in the hardness of oxidized wax (Figure 1) [27]. Analytically pure calcium hydroxy is employed for the calcification reaction experiment, with its main reaction formula represented as follows in (1)

(1) $$C_{n}H_{2n}O_{2}+Ca{\left(OH\right)}_{2}\to C_{n}H_{2n-2}O_{2}Ca+H_{2}O$$

Preparation method: Place 100 g of BW, RBW, and CW into separate beakers with a volume of 500 mL and heat them until completely melted, stirring using magnetic force. Once the temperature reaches approximately 120 °C, introduce the pre-weighed *Ca*(*OH*)_2_ for calcification modification (wax to calcium ratio: 5:5, 6:4, 7:3, 8:2, or 9:1), followed by multiple washes and steam treatments.

### 4.4. Preparation of KPSO Gel

KPSO oil gel was prepared using uncalcified wax BW, RBW, CW, and calcified wax (BW:Ca, RBW:Ca, CW:Ca) as gel agents, respectively. Different dosages of gels were investigated for the preparation conditions. A total of 5 g of KPSO was weighed, and gel doses of 3%, 5%, 7%, and 11 wt% by weight were added, respectively. The stirring and heating time was set at 25 min. The optimal concentration for gelatinization at temperature was studied, and the overall texture and microstructure were analyzed. Subsequently, the samples were cooled to a temperature of 4 °C and kept at this temperature for a duration of 12 h before being stored at a temperature of 20 °C. All samples were analyzed after being stored at a temperature of 20 °C for a period of 48 h.

### 4.5. Characterization of KPSO Gel Properties

#### 4.5.1. Critical Gelling Ability (C*) Analysis

The uncalcified wax concentration was evaluated at two different concentration ranges [3,20,22]. Initially, wax (BW, RBW, CW) was incrementally added at concentrations of 1, 2, 3, 4, and 5 wt% to assess gel formation. Subsequently, concentrations of 0.5, 1.5, 2.5, 3.5, and 4.5 wt% were employed to determine the presence of gel oil. The specific methodology involved adding a volume of melted gel oil sample (6 mL) into a vial with a capacity of 10 mL, followed by reformation of the gel at a temperature of 5 °C. All test tubes were then inverted for 1 h at a temperature of 25 °C until no visible flow was observed in the vial; this indicated the critical gel concentration.

#### 4.5.2. Texture Profile Analysis

The KPSO gel was evaluated using Brookfield’s CT3 Texture profile analysis [12,13], employing a cylindrical probe with a diameter of 0.5 mm (*p* = 0.05) for texture key experimental parameters included speed of 2.0 mm/s, test 2.0 mm/s, post-test speed of 5.0 mm/s, automatic trigger type, and trigger force set at 5 g. Three parallel experiments were carried out on each sample to evaluate the hardness, elasticity, cohesion, adhesion, and chewability properties of KPSO gels.

#### 4.5.3. Analysis of Temperature Intervention Gel Strength Change

Oil-gel phase maps of KPSO were constructed using uncalcified wax and calcified wax, respectively, enabling visual observation and comparison of the changes in dissolution temperatures between the two groups. The storage temperature range for the experiment was set at 50 to 90 °C, with a 10 °C interval, allowing for the determination of the melting point variation. They were left at each cooling temperature (25, 15, 5, 0 °C) for 24 h. The hardness test was conducted by 5.5.2TPA, and the relationship between the influence of cooling temperature and hardness was discussed [3].

#### 4.5.4. Oil-Holding Capacity Measurement

The oil-holding capacity of KPSO gel was determined using a D5A centrifuge (Hunan Kaida Scientific Instrument Co., Ltd. (Changsha, China)) [21]. Approximately 1 g of the oil gel sample was subjected to centrifugation at 10,000 rpm for 15 min, followed by inversion of the centrifuge tube to remove any excess oil. The combined oil capacity is calculated as follows in (2), where *m*_1_ represents the total mass of the initial sample and centrifugal tube, *m*_2_ denotes the total mass of the sample and centrifugal tube after removing oil loss (OL), and m refers to the mass of the centrifugal tube.

(2) $$OBC\left(\%\right)=\frac{\left(m_{1}-m\right)-\left(m_{2}-m\right)}{m_{1}-m}$$

#### 4.5.5. Determination of Molecular Forces by Fourier Spectroscopy

The US Platinum Elmer IS-10 Fourier infrared spectroscopy, equipped with an Attenuated Total Reflection (ATR) sampling attachment, was employed to obtain the spectrum of the oil gel sample in the wavelength range of 400~4000 cm^−1^ [4]. The obtained spectrum underwent air background subtraction and analysis of intermolecular forces using OMNIC (Thermo, v8.0) software.

#### 4.5.6. Micro Structure Analysis

The crystal microstructure and morphology of the sample were analyzed using a ZEISS Primostar3 microscope from ZEISS, Germany. After melting at 90 °C for 30 min, all samples were stored in incubators at either 4 °C or 20 °C for observation for 48 h. The fractal dimension (Db) and mean crystal size [22] were spatially precalibrated using the scale on the image. Particle size distribution and average particle size were determined for each sample by analyzing 25–35 particles using Image J software v.1.49 (NIH, Bethesda, Rockville, MD, USA), following the research methods described by Winkler-Moser al. [11].

### 4.6. Construction of Digestive Model In Vitro

The in vitro digestion model consisted of three successive stages [31,32,34,35]. For the oral stage, 4 g of KPSO gel was mixed with 4 g of artificial saliva and heated in a water bath at 37 °C for 1 min. In the gastric phase, 20 g of gastric juice (3.2 g/L pepsin) was adjusted to pH 2 using both 1 M and 6 M HCl. The sample was then incubated in an oscillating water bath at a constant pH of 2.00 ± 0.02 for 1 h [41,42]. For the intestinal phase, the pH of the gastric chyme was adjusted to 7 using NaOH solutions with concentrations of 0.1 M, 1 M, and 10 M, respectively. Subsequently, 30 g of intestinal fluid was added to 10 g of oil gel containing chyme that included 150 mM NaCl, 10 mM CaCl_2_, 6 mg/mL bile salts, and 1.5 mg/mL pancreatic enzymes [24,43]. The digestion process was evaluated within 2 h using a METTler Toledo Instruments (Shanghai) pH static titrator, with 0.05 M NaOH as the titrant, and the endpoint was determined at pH 7.0. A small portion of the digested mixture was examined under a 400-fold optical microscope after being applied to a glass rod [44,45,46].

#### 4.6.1. Simulated Oral Digestion

The oral digestive solution, with a pH of 6.8 ± 0.2, containing α-amylase was prepared. It ultimately comprised of 0.896 g/L KCl, 0.2 g/L KSCN, 0.888 g/L NaH_2_PO_4_, 0.57 g/L Na_2_SO_4_, and 0.298 g/L NaCl. Additionally, the solution contained concentrations of 1.694 g/L NaHCO_3_, 0.2 g/L urea, and 0.015 g/L uric acid while maintaining an α-amylase concentration of 0.6 g/L. The oil gel sample (4 g) was thoroughly mixed with an oral digestive solution containing alpha-amylase (4 mL). Subsequently, the pH of the mixture was adjusted to 6.8 ± 0.2, and the temperature was maintained at 37 °C. Stirring the mixture at a speed of 100 rpm for 3 min ensued [41,42].

#### 4.6.2. Simulated Gastric Digestion

The simulated gastrointestinal digestive fluid was prepared by dissolving 2 g NaCl, 7 mL HCl, and 3.2 g pepsin in distilled water, followed by adjusting the volume to 1 L with distilled water. After thorough shaking, the pH of the simulated gastric fluid was adjusted to 1.2 using a 0.5 mol/L HCl solution. For the simulated digestion process, a mixture containing oral digestion products was supplemented with 20 mL of simulated gastric juice, and its pH was adjusted to 2.5 using a 0.5 mol/L NaOH solution before being digested at 37 °C on a shaking table set at 110 rpm for 1 h. A suitable amount of partially digested material was then sampled for characterization analysis [43]

#### 4.6.3. Simulated Intestinal Digestion

The simulated intestinal fluid was prepared with a concentration of 12.0 mg/mL bile salts, 2.0 mg/mL pancreatic enzymes, 6.8 mg/mL KH_2_PO_4_, and 8.8 mg/mL NaCl. We precisely collected 20 mL of the remaining gastric digestion sample solution and added it to 20 mL of simulated intestinal fluid, ensuring thorough mixing of the two components. The resulting mixture was incubated for 2 h at a temperature of 37 °C on a shaker, while pH measurements were taken at intervals of 30, 60, 90, and 120 min. To maintain a pH value within the range of 7.0 ± 0.2 during the experiment, adjustments were made using a solution of 0.l mol/L NaOH as necessary; the volume consumed was recorded accordingly. Finally, the concentration of free fatty acids generated through Mit-lipo was calculated based on the volume of NaOH consumed using Formula (3) [44,45].

(3) $$FFA\%=100\times \frac{V\times m\times M_{lipid}}{w_{lipid}\times 2}$$

where *V*: volume of sodium hydroxide consumed (mL); *M_lipid_*: molecular weight of oil; *W_lipid_*: the weight of the digestive system by oil (g).

### 4.7. Statistical Analysis

All experiments were performed in triplicate, the results are expressed as the mean ± standard deviation, and statistical analysis was completed using Origin2018. One-way analysis of variance (ANOVA) and Waller–Duncan test (*p* < 0.05) were analyzed using the SPSS 26.0 statistical software program.

## 5. Conclusions

In this study, a self-responsive, environmentally adaptable calcified wax KPSO gel was prepared by a one-step method by adjusting the proportions of three kinds of wax and calcium. The results show that compared with the uncalcified wax, the same van der Waals force is the main molecular force, but the hardness of the oil gel formed by calcified wax is significantly reduced, and the melting point is also increased by about 10 °C. In addition, a higher Ca^2+^ ratio leads to a higher nucleation rate. Moreover, the increase in Ca^2+^ significantly inhibits the release of lipids from the oil gel during the simulated digestion process in vitro. These findings may help in the development of products rich in unsaturated fats to promote health and reduce the risk of disease associated with fat consumption.

## Figures and Tables

**Table 1:** Texture and physical properties of KPSO Gel (25 °C).

Sample		Hardness (g)	Adhesive Force (g)	Cohesiveness (mj)	Elasticity (mm)	Adhesion (g)	Mastication (mj)
Group1							
BW		1479.04 ± 6.04 ^a^	373.33 ± 10.12 ^a^	0.72 ± 0.11 ^b^	6.77 ± 1.39 ^a^	14.67 ± 0.59 ^c^	43.36 ± 1.28 ^a^
BW:Ca	BW5:Ca5	112.18 ± 4.22 ^f^	5.51 ± 0.51 ^ef^	0.13 ± 0.02 ^ef^	0.43 ± 0.02 ^e^	13.33 ± 1.53 ^d^	0.38 ± 0.07 ^e^
	BW6:Ca4	529.10 ± 14.55 ^b^	153.53 ± 8.72 ^b^	0.13 ± 0.06 ^e^	1.76 ± 0.11 ^c^	33.83 ± 1.35 ^a^	0.46 ± 0.14 ^c^
	BW7:Ca3	480.01 ± 5.02 ^c^	18.19 ± 0.25 ^d^	0.25 ± 0.06 ^d^	0.12 ± 0.02 ^f^	12.33 ± 0.43 ^e^	0.43 ± 0.04 ^d^
	BW8:Ca2	268.10 ± 7.40 ^e^	5.86 ± 5.80 ^e^	1.92 ± 2.52 ^a^	3.20 ± 4.49 ^b^	20.25 ± 9.74 ^b^	14.53 ± 14.35 ^b^
	BW9:Ca1	340.38 ± 15.79 ^d^	116.33 ± 0.47 ^c^	0.61 ± 0.48 ^c^	1.64 ± 0.50 ^d^	9.92 ± 0.09 ^f^	0.17 ± 0.03 ^f^
Group2							
RBW		143.26 ± 16.68 ^f^	25.00 ± 2.06 ^a^	0.31 ± 0.31 ^f^	0.52 ± 0.06 ^e^	17.09 ± 0.93 ^e^	0.22 ± 0.14 ^f^
RBW:Ca	RBW5:Ca5	269.04 ± 15.29 ^d^	15.33 ± 0.47 ^e^	0.90 ± 0.05 ^a^	0.87 ± 0.11 ^a^	27.92 ± 1.09 ^b^	0.65 ± 0.43 ^a^
	RBW6:Ca4	258.59 ± 37.97 ^e^	16.97 ± 0.31 ^d^	0.62 ± 0.19 ^b^	0.69 ± 0.26 ^b^	46.42 ± 0.60 ^a^	0.46 ± 0.36 ^d^
	RBW7:Ca3	318.46 ± 9.18 ^c^	14.09 ± 3.14 ^f^	0.49 ± 0.23 ^e^	0.65 ± 0.30 ^c^	26.22 ± 10.68 ^c^	0.54 ± 0.24 ^c^
	RBW8:Ca2	327.33 ± 6.14 ^b^	19.19 ± 0.25 ^c^	0.53 ± 0.32 ^d^	0.46 ± 0.07 ^f^	18.81 ± 5.45 ^d^	0.61 ± 0.20 ^b^
	RBW9:Ca1	365.22 ± 7.98 ^a^	22.42 ± 2.68 ^b^	0.54 ± 0.27 ^c^	0.63 ± 0.23 ^d^	16.83 ± 1.82 ^f^	0.45 ± 0.16 ^e^
Group3							
CW		1066.92 ± 24.32 ^a^	253.67 ± 10.69 ^a^	0.31 ± 0.31 ^bc^	3.28 ± 0.26 ^a^	65.09 ± 2.60 ^a^	5.25 ± 0.27 ^a^
CW:Ca	CW5:Ca5	33.92 ± 1.24 ^d^	3.06 ± 0.24 ^e^	0.54 ± 0.30 ^ab^	0.36 ± 0.03 ^d^	7.59 ± 0.34 ^e^	0.18 ± 0.06 ^f^
	CW6:Ca4	14.99 ± 0.36 ^e^	1.26 ± 0.20 ^f^	0.36 ± 0.40 ^b^	0.33 ± 0.19 ^e^	5.47 ± 0.33 ^f^	0.67 ± 0.14 ^b^
	CW7:Ca3	126.68 ± 13.82 ^b^	5.12 ± 0.10 ^b^	0.05 ± 0.03 ^d^	0.26 ± 0.17 ^f^	13.22 ± 2.43 ^d^	0.37 ± 0.01 ^e^
	CW8:Ca2	120.46 ± 7.58 ^bc^	4.39 ± 0.34 ^c^	0.15 ± 0.13 ^c^	0.51 ± 0.14 ^c^	19.81 ± 4.90 ^c^	0.48 ± 0.31 ^d^
	CW9:Ca1	69.89 ± 2.77 ^c^	3.36 ± 0.31 ^d^	0.77 ± 0.14 ^a^	1.43 ± 0.41 ^b^	31.31 ± 2.05 ^b^	0.62 ± 0.27 ^c^

Note: Values are presented as the mean ± standard deviation of three replicates. The lowercase letters within the same column indicate significant differences (*p* < 0.05).

**Table 2:** The average particle size of KPSO gels of different proportions.

Samples		Effect of Wax Base Concentration (wt%) on Average Particle Size (μm)				
		3 wt%	5 wt%	7 wt%	9 wt%	11 wt%
Group1						
BW		6.56 ± 0.37 ^b^	7.67 ± 0.31 ^a^	8.50 ± 0.45 ^a^	9.56 ± 0.36 ^a^	11.18 ± 0.69 ^a^
BW:Ca	BW5:Ca5	5.08 ± 0.57 ^d^	4.96 ± 0.60 ^d^	5.08 ± 0.50 ^e^	4.97 ± 0.68 ^f^	4.63 ± 0.30 ^e^
	BW6:Ca4	5.57 ± 0.35 ^c^	5.60 ± 0.31 ^c^	5.62 ± 0.33 ^d^	5.50 ± 0.30 ^e^	5.96 ± 0.59 ^d^
	BW7:Ca3	6.56 ± 0.33 ^b^	6.96 ± 0.58 ^b^	6.48 ± 0.41 ^c^	6.45 ± 0.28 ^d^	6.43 ± 0.25 ^c^
	BW8:Ca2	7.53 ± 0.36 ^b^	7.02 ± 0.46 ^b^	6.56 ± 0.38 ^c^	6.71 ± 0.29 ^c^	6.57 ± 0.31 ^c^
	BW9:Ca1	7.52 ± 0.31 ^a^	7.98 ± 0.57 ^a^	8.36 ± 0.98 ^b^	9.01 ± 0.66 ^b^	9.58 ± 0.79 ^b^
Group2						
RBW		7.49 ± 0.32 ^a^	7.86 ± 0.53 ^a^	8.62 ± 0.97 ^a^	8.43 ± 0.95 ^a^	8.30 ± 0.78 ^a^
RBW:Ca	RBW5:Ca5	5.96 ± 0.53 ^d^	6.95 ± 0.58 ^b^	6.92 ± 0.57 ^c^	6.47 ± 0.40 ^cd^	5.98 ± 0.62 ^d^
	RBW6:Ca4	6.95 ± 0.52 ^c^	6.11 ± 0.55 ^d^	5.89 ± 0.54 ^e^	6.22 ± 0.56 ^d^	6.26 ± 0.57 ^d^
	RBW7:Ca3	7.24 ± 0.47 ^ab^	7.09 ± 0.65 ^b^	6.08 ± 0.65 ^e^	6.70 ± 0.96 ^bc^	6.65 ± 0.72 ^c^
	RBW8:Ca2	6.10 ± 0.60 ^d^	6.52 ± 0.86 ^c^	6.55 ± 0.74 ^d^	6.93 ± 0.60 ^b^	7.14 ± 0.55 ^b^
	RBW9:Ca1	7.08 ± 0.59 ^bc^	7.12 ± 0.55 ^b^	7.48 ± 0.86 ^b^	6.27 ± 0.89 ^d^	7.07 ± 0.67 ^b^
Group3						
CW		7.07 ± 0.63 ^b^	6.51 ± 0.88 ^cd^	6.47 ± 0.34 ^cd^	6.20 ± 0.67 ^c^	6.98 ± 0.60 ^a^
CW:Ca	CW5:Ca5	7.24 ± 1.14 ^b^	6.09 ± 0.61 ^e^	6.17 ± 0.69 ^d^	6.66 ± 0.94 ^b^	7.08 ± 0.65 ^a^
	CW6:Ca4	7.00 ± 0.65 ^b^	6.89 ± 0.54 ^ab^	6.80 ± 0.98 ^bc^	6.63 ± 0.86 ^bc^	6.22 ± 0.56 ^b^
	CW7:Ca3	6.17 ± 0.70 ^c^	6.57 ± 0.35 ^bc^	7.05 ± 0.57 ^ab^	6.95 ± 0.65 ^b^	6.48 ± 0.92 ^b^
	CW8:Ca2	7.12 ± 0.56 ^b^	6.16 ± 1.19 ^de^	7.03 ± 0.64 ^ab^	6.62 ± 1.03 ^bc^	5.65 ± 1.36 ^c^
	CW9:Ca1	8.58 ± 0.33 ^a^	7.07 ± 0.48 ^a^	7.47 ± 0.98 ^a^	7.51 ± 0.88 ^a^	7.21 ± 1.19 ^a^

Note: Values are shown as the mean ± standard deviation of 25 replicates. The values of different lowercase letters in the same column are significantly different (*p* < 0.05). Particle size data are in Appendix A.

**Table 3:** Average particle size of KPSO gel after simulated digestion (oral, gastric, and intestinal) in vitro.

Sample		Average Particle Size/μm		
		Oral	Gastric	Intestinal
Group1				
BW		10.99 ± 0.60 ^a^	10.03 ± 0.61 ^a^	10.47 ± 0.39 ^a^
BW:Ca	BW5:Ca5	5.04 ± 0.61 ^d^	6.50 ± 0.28 ^c^	6.19 ± 0.65 ^c^
	BW6:Ca4	5.89 ± 0.69 ^c^	5.04 ± 0.53 ^f^	5.56 ± 1.09 ^d^
	BW7:Ca3	5.66 ± 0.32 ^c^	6.00 ± 0.66 ^d^	5.95 ± 0.75 ^c^
	BW8:Ca2	5.95 ± 0.62 ^c^	5.58 ± 0.27 ^e^	5.54 ± 0.97 ^d^
	BW9:Ca1	9.33 ± 0.96 ^b^	8.82 ± 1.30 ^b^	9.20 ± 0.57 ^b^
Group2				
RBW		8.08 ± 0.67 ^a^	7.48 ± 0.33 ^a^	7.06 ± 0.54 ^a^
RBW:Ca	RBW5:Ca5	6.54 ± 0.30 ^c^	5.45 ± 0.42 ^d^	5.57 ± 0.90 ^d^
	RBW6:Ca4	6.44 ± 0.89 ^c^	5.99 ± 0.74 ^c^	6.64 ± 0.37 ^b^
	RBW7:Ca3	6.54 ± 0.34 ^c^	6.16 ± 0.69 ^c^	5.64 ± 0.35 ^d^
	RBW8:Ca2	7.15 ± 0.55 ^b^	7.10 ± 0.64 ^b^	6.03 ± 0.55 ^c^
	RBW9:Ca1	7.04 ± 0.54 ^b^	6.21 ± 0.60 ^c^	6.71 ± 0.84 ^b^
Group3				
CW		6.43 ± 0.37 ^c^	6.50 ± 0.36 ^a^	6.55 ± 0.40 ^a^
CW:Ca	CW5:Ca5	6.51 ± 0.33 ^c^	5.97 ± 0.71 ^b^	6.04 ± 0.70 ^b^
	CW6:Ca4	6.48 ± 0.31 ^c^	6.06 ± 0.53 ^b^	5.58 ± 0.32 ^c^
	CW7:Ca3	6.55 ± 0.37 ^c^	6.12 ± 0.69 ^b^	6.13 ± 1.15 ^bc^
	CW8:Ca2	7.13 ± 0.63 ^b^	6.18 ± 0.70 ^ab^	5.98 ± 0.60 ^bc^
	CW9:Ca1	7.44 ± 0.91 ^a^	6.54 ± 0.34 ^a^	6.66 ± 1.07 ^a^

Note: values are shown as the mean ± standard deviation of 25 replicates. The values of different lowercase letters in the same column are significantly different (*p* < 0.05); particle size data are in Appendix B.

**Figure A1:**
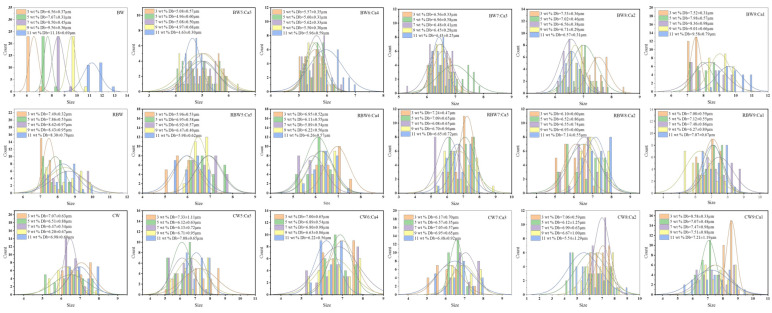
Distribution of mean particle size of KPSO gels of different proportions.

**Figure A2:**
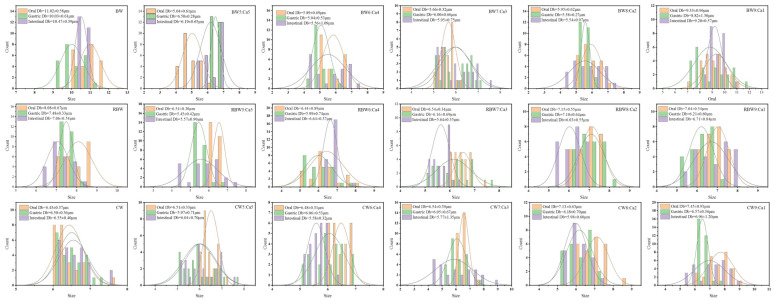
Distribution of mean particle size of KPSO gel following in vitro simulated digestion (oral, gastric, and intestinal phases).

## Data Availability

All available data are contained within the article.

## Abbreviations

Korean pine seed oil, KPSO; Free Fatty Acids, FFA; Beeswax, BW; Rice bran wax, RBW; Carnauba wax, CW. Fourier transform infrared spectroscopic, *FT-IR*; Fractal dimension, Db.
